# ASC specks exacerbate α‑synuclein pathology via amplifying NLRP3 inflammasome activities

**DOI:** 10.1186/s12974-023-02709-w

**Published:** 2023-02-05

**Authors:** Ran Zheng, Yiqun Yan, Shaobing Dai, Yang Ruan, Ying Chen, Chenjun Hu, Zhihao Lin, Naijia Xue, Zhe Song, Yi Liu, Baorong Zhang, Jiali Pu

**Affiliations:** 1 Department of Neurology, School of Medicine, Second Affiliated Hospital, Zhejiang University, Hangzhou, 310009 Zhejiang China; 2 Department of Anesthesiology, School of Medicine, Women’s Hospital, Zhejiang University, Hangzhou, 310009 Zhejiang China; 3 Department of Human Anatomy, Histology and Embryology, System Medicine Research Center, Zhejiang University School of Medicine, Hangzhou, 310058 Zhejiang China

**Keywords:** NLRP3 inflammasome, ASC specks, Microglia, α‑synuclein pathology, Parkinson’s disease

## Abstract

**Background:**

Inflammasome activation has a pathogenic role in Parkinson’s disease (PD). Up-regulated expressions of inflammasome adaptor apoptosis-associated speck-like protein containing a CARD (ASC) and assembly of ASC specks have been observed in postmortems of human PD brains and experimental PD models. Extracellular ASC specks behave like danger signals and sustain prolonged inflammasome activation. However, the contribution of ASC specks in propagation of inflammasome activation and pathological progression in PD has not been fully established.

**Methods:**

Herein, we used human A53T mutant α-synuclein preformed fibrils (PFFs)-stimulated microglia in vitro and unilateral striatal stereotaxic injection of PFFs-induced mice model of PD in vivo, to investigate the significance of ASC specks in PD pathological progression. Rotarod and open-field tests were performed to measure motor behaviors of indicated mice. Changes in the molecular expression were evaluated by immunofluorescence and immunoblotting (IB). Intracellular knockdown of the ASC in BV2 cells was performed using si-RNA. Microglial and neuronal cells were co-cultured in a trans-well system to determine the effects of ASC knockdown on cytoprotection.

**Results:**

We observed a direct relationship between levels of ASC protein and misfolded α‑synuclein aggregates in PD mice brains. ASC specks amplified NLRP3 inflammasome activation driven by α-synuclein PFFs stimulation, which aggravated reactive microgliosis and accelerated α‑synuclein pathology, dopaminergic neurodegeneration and motor deficits. Endogenous ASC knockdown suppressed microglial inflammasome activation and neuronal α‑synuclein aggregation.

**Conclusions:**

In conclusion, our study elucidated that ASC specks contribute to the propagation of inflammasome activation-associated α‑synuclein pathology in PD, which forms the basis for targeting ASC as a potential therapy for PD.

**Supplementary Information:**

The online version contains supplementary material available at 10.1186/s12974-023-02709-w.

## Background

Globally, Parkinson’s disease (PD) is the second leading cause of neurodegeneration, affecting 2–3% of the population aged over 65 years [1]. The pathophysiology of PD is characterized by progressive degeneration of nigrostriatal dopaminergic neurons and intracellular accumulation of aggregates containing misfolded fibrillar α‑synuclein in the remaining neurons [2]. Even though several etiological theories for PD have been proposed [3], the underlying molecular pathogenesis mechanisms have not been fully defined.

Recently, the interplay between inflammation and misfolded protein aggregates has gained attention to explain the mechanism of neurodegenerative disease progression [4]. The inflammation-based hypothesis suggested that inflammatory response driven by activated microglia contributes to protein misfolding and aggregation, and that aggregates enhance neuroinflammation in a positive feedback manner as the disease progresses, ultimately leading to neurodegeneration [5].

Notably, inflammasome activation is one of the most well characterized inflammatory pathogenic factors in PD [6–8]. In particular, activation of NLR family pyrin domain-containing 3 (NLRP3) inflammasome, a central intracellular sensor of danger signals, has been documented in post-mortems of human PD brains [9, 10]. NLRP3 Inflammasome activation relies on two signals: transcriptional upregulation of inflammasome components via signal 1 for priming, and inflammasome assembly via signal 2 for activation [11, 12]. Upon activation, the NLRP3 inflammasome recruits effector pro-inflammatory cysteinyl aspartate-specific proteinases (caspases) via adaptor apoptosis-associated speck-like protein containing a CARD (ASC), followed by maturation of caspases and secretion of inflammatory cytokines [13, 14]. Gasdermin D (GSDMD) is an effector for pyroptosis downstream of the inflammasome signaling pathways [15, 16]. Cleavage of GSDMD by caspases allows its N-terminal domain interacts with membrane lipids and to assemble pores that contribute to the release of mature cytokines [17, 18]. Interestingly, the adaptor ASC is a bipartite protein composed of a PYD domain and a CARD domain. Compelling evidence indicates that PYD and CARD domains self-associate and tend to form dense, crosslinked filamentous structures so-called ASC specks [19–21], which exhibit a seeding behavior or a propensity to aggregate in a prion-like manner [22, 23]. The assembled ASC specks can be taken up by adjacent myeloid cells after release into the intercellular space and behave like danger signals, thereby sustaining prolonged inflammasome activation [22, 24, 25]. Although numerous studies have established that NLRP3 inflammasome activation drives or promotes misfolded protein aggregation [9, 26, 27], the exact role of ASC in pathological progression remains unclear, as ASC is more than a mere inflammasome adapter [28].

In APP/PS1 Alzheimer’s disease (AD) mice models, microglia-derived ASC specks acted as cross-seeds for amyloid-β pathology, resulting in the spread of amyloid-β pathology [29]. This composite of ASC and amyloid-β amplified the activation of NLRP3 inflammasomes and release of ASC specks, which started a vicious cycle of vast inflammasome activation [30]. In addition, ASC inflammasome could modulate exogenously and non-exogenously seeded Tau pathology, whereas ASC deficiency decreased Tau pathology in Tau transgenic mice [31]. These findings have opened the door to a new paradigm for studying inflammasome-related neurodegenerative pathogenesis.

In PD, studies using immortalized microglial cell lines and human microglia have reported that insoluble α‑synuclein aggregates can trigger inflammasome activation similar to amyloid-β [10, 32, 33]. Up-regulated ASC levels and formation of ASC specks have also been observed in the brains of PD patients and preclinical PD models [9]. In addition, a welder population exposed to Mn exhibited a dose-dependent progression of Parkinsonian syndrome, had higher loads of ASC and inflammatory cytokine levels in serum exosomes and serum [34]. Compared to controls, gene expression levels of ASC have been found to be elevated in peripheral blood mononuclear cells (PBMCs) of PD patients, while plasma levels of NLRP3 and IL-1β exhibited a positive correlation with motor severity in PD patients [35, 36].

Given the above evidence and clues, we investigated the interplay between ASC specks formation, NLRP3 inflammasome activation and pathological progression in human A53T mutant α-synuclein preformed fibrils (PFFs)-induced PD models. The A53T mutation is key in defining the differences in the aggregation kinetics of human and mouse α-synuclein [37], and A53T mutant PFFs induce neuroinflammation in vivo more rapidly than wildtype (WT) PFFs [38, 39]. We demonstrated a direct relationship between ASC expression levels and α‑synuclein pathology as the disease progresses. The ASC specks amplified NLRP3 inflammasome activation in PD models, which exacerbated microglial dysfunction, α‑synuclein accumulation and dopaminergic neurodegeneration. Targeting ASC may serve as a promising therapeutic strategy to inhibit the pathological progression of PD.

## Methods and materials

### Chemicals and reagents

Dulbecco's Modified Eagle’s Medium (DMEM), fetal bovine serum (FBS) and other cell culture reagents, goat anti-mouse- AlexaFluor594 (H + L) antibody, goat anti-rabbit-AlexaFluor488 (H + L) antibody and rabbit IgG isotype control antibody were obtained from Thermo Fisher Scientific (Invitrogen). si-m-PYCARD/ASC RNAs were obtained from Guangzhou RiboBio Co., Ltd. Mouse anti-NLRP3 (Cat# AG-20B-0014), mouse anti-caspase-1 (Cat# AG-20B-0042) and rabbit anti-ASC (Cat# AG-25B-0006) were purchased from AdipoGen Life Sciences. Rabbit anti-α-synuclein (Cat# 4179), rabbit anti-Phospho-α-synuclein (Cat# 23706), rabbit anti-phospho-NF-κB p65 (Cat# 3033) and mouse anti-IL-1β (Cat# 12242) were purchased form Cell Signaling Technology. Rabbit anti-ASC (Cat# YT0365) was purchased from ImmunoWay Biotechnology. Mouse anti-Phospho-α-synuclein (Cat# pSyn #64) and rabbit anti-IBA1 (Cat# 019-19741) were purchased from FUJIFILM Wako Pure Chemical Corporation. Mouse anti-α-synuclein (Cat# sc-12767) and mouse anti-GSDMD (Cat# sc-393581) was purchased from Santa Cruz Biotechnology. Rabbit anti-NF-κB p65 (Cat# ab16502), goat anti-IBA1 (Cat# ab5076) and rabbit anti-GSDMD (Cat# ab219800) were purchased from Abcam. Rabbit anti-IL18 (Cat# A1115) was purchased from ABclonal. Rabbit anti-tyrosine hydroxylase (Cat# AB152) was purchased from Merck Millipore.

### Generation of recombinant human A53T mutant α-synuclein PFFs

Recombinant human A53T mutant α-synuclein PFFs from HD Biosciences (Shanghai, China) were generated to a final concentration of 1.45 mg/ml (~ 100 μM) and a final purity of 90%. Briefly, the human A53T α-synuclein protein coding region was sub-cloned into a pET-24a expression vector. After transformation to *E. coli* BL21 (DE3), expression levels of target proteins were confirmed by immunoblotting (IB) assays. The Co-NTA agarose column was used to purify the expressed A53T α-synuclein protein while the Thioflavin T (ThT) assay was used to monitor the aggregation process. PFFs were aliquoted and stored at − 80 °C. Immediately before intracerebral injections, PFFs aliquots were thawed at room temperature and sonicated in an ultrasonic bath sonicator (Diagenode Biorupter Pico sonication device) for 15 cycles of 30 s on and 30 s off.

### Preparation of ASC specks

Recombinant ASC from Huabio (Hangzhou, China) was generated as previously described, with minor modifications [30]. Briefly, full-length human ASC was cloned in NdeI/XhoI sites of a pET-23a expression vector providing a His tag. After transformation to *E. coli* BL21 (DE3), *E. coli* cells were incubated at 37 °C and induced with 1 mM isopropyl b-D-1-thiogalactopyranoside (IPTG) and shaken at 220 rpm for 4 h. Then, cells were obtained and lysed with sonication. Cell lysates were centrifuged after which the pellet was resuspended. After centrifugation, the supernatant was administered to a pre-equilibrated Ni-TED beads column. The purified protein was dialysed under continuous stirring and condensed to a final concentration of 0.5 mg/ml (~ 20 μM). The purified protein was validated by running the samples in 12% SDS-PAGE followed by immunoblotting using an anti-ASC antibody. To induce fibrillation, the ASC-containing solution was transformed to LoBind Tubes (Eppendorf) and incubated for 1 h at 37 °C. Before use, the ASC specks were kept at 4 °C for no longer than 3 weeks.

### Transmission electron microscopy (TEM)

The ASC specks and human A53T mutant α-synuclein PFFs samples were characterized by TEM. For negative staining electron microscopy, samples were diluted in phosphate buffered saline (PBS) to a final concentration of 0.1 mg/ml. The diluted solutions were adsorbed to 300 mesh copper grids for 5 min at RT, and negatively stained with 2% uranyl acetate for 1 min. Imaging was performed at × 205,000 magnification using a FEI Tecnai 10 transmission electron microscope at 100 kV equipped with a TEM side-mounted camera.

### Animals and stereotaxic injections

Adult male C57BL/6 mice (8 weeks old) were obtained from SLAC Laboratory Animal Co., Ltd. (Shanghai, China), acclimatized for 1 week, randomized into different experimental groups and anesthetized using 0.9% pentobarbital (0.1 ml/g i.p.) before surgical procedures. Each mouse received a unilateral (right) intrastriatal injection of an equal volume of reagent (see details in Fig. 3 and Additional file 1: Fig. S3). Stereotaxic striatal injections coordinates from the bregma were: anterior–posterior (AP) + 0.4 mm; medial–lateral (ML)—2.0 mm; dorsal–ventral (DV) -3.5 mm at an infusion rate of 0.4 μl/min. After surgery, mice were monitored until awake and returned to the cages. Six- or twelve-weeks post-surgery, mice were subjected to behavioral tests and sacrificed.

### Behavioral training and tests

Six or twelve weeks after surgery, mice were challenged via open field and rotarod tests to valuate motor functions. Rotarod tests were performed as previously described, with minor modifications [40]. Briefly, mice were trained to run on the rotating rod for three consecutive days, prior to the final test. After training, mice were placed on the rotating rod with the rotation speed gradually increased from 4 to 40 rpms within a 5 min period. The duration that mice remained on the rod until their first slip to the base (also called latency to falling) was recorded. Measurements were averaged over three trials, with at least 1 h of rest. To assess the general locomotor activities and exploratory behaviors, mice were tested in the open field task for 5 min without disturbance. Movements were monitored with a camera linked to a SMART video tracking software (Smart 3.0) as the animals moved around in a cubic box. The total distance traveled and the distance in middle zone were recorded and analyzed for each mouse.

### Isolation of primary microglia

To obtain primary microglial cells, cerebral cortices of newborn Sprague Dawley rats (postpartum day 0–2) were collected and incubated at 37 °C for 15 min in 0.25% trypsin [41]. After centrifugation, pellets were lysed into a cell suspension and filtered via a 70-μm pore size filter. Cells of two cortices were seeded in a poly-D-lysine (PDL)-coated T-75 culture flask containing warm DMEM supplemented with 10% FBS and 1% penicillin‒streptomycin to generate mixed glial cultures. The entire culture medium was replaced after 24 h, and half of the medium replaced every 3–5 days. On day 10, the microglia were stimulated using 25 ng/ml colony-stimulating factor (CSF). Three days after stimulation, they were detached from mixed glial cultures under shaking at 200 rpm for 2 h at 37 °C. The obtained microglia were seeded in 12-well plates for further treatment.

### Cell culture and transfection

Mouse BV2 cells and human SH-SY5Y cells were obtained from the Cell Bank of the Chinese Academy of Sciences (Beijing, China) and cultured in DMEM supplemented with 10% FBS in 5% CO_2_ at 37 °C. ASC knockdown in BV2 cells was performed using si-PYCARD/ASC and an RNAiMAX transfection reagent (Thermo Fisher Scientific), according to the manufacturer’s protocol. At 48 h post-transfection, cells lysates were collected for IB to verify the transfection efficiencies of knockdown. To determine the effects of ASC knockdown in BV2 cells on SH-SY5Y cells, SH-SY5Y cells in the bottom and BV2 cells on top were separated in a trans-well culture with 0.4 μm pore polyester membrane inserts (Corning cat# 3470) in co-culture conditions. BV2 cells with or without ASC gene knockdown were stimulated with PFFs after which cell lysates and supernatants were collected after 16 h of co-culture.

### Immunofluorescence

To perform immunofluorescence of brain slices, mice were anesthetized and thereafter perfused using PBS, followed by 4% paraformaldehyde (PFA). The brains were dissected, fixed in 4% PFA solution for 6 h at 4 °C, and dehydrated in at least 10-times the brain volume of 30% sucrose solution until the brains sunk to the bottom. Then, the brains were embedded in an OCT compound (Sakura) and serially sliced into 30 μm thick sections using a cryostat microtome (Leica CM1950). After blocking and permeabilization, brain slices were incubated in the presence of primary antibodies at 4 °C overnight. On the next day, slices were washed thrice using PBS, incubated with fluorescent secondary antibodies for 2 h at RT, washed thrice, supplemented with anti-fading agents containing 4,6-diamidino-2-phenylindole (DAPI) and mounted.

Immunofluorescence of BV2 cells, primary microglial cells and SH-SY5Y cells were performed after treatment. Cells were fixed for 20 min in 4% PFA at RT and incubated for 30 min with 5% BSA and 0.3% Triton X‐100 at 37 °C for blocking and permeabilization. After overnight incubation in the presence of primary antibodies at 4 °C, cells were washed and incubated with fluorescent secondary antibodies for 2 h at RT. Then, coverslips were washed and subsequently incubated with anti-fading agents containing DAPI for 5 min and mounted on glass slides. Images were acquired by laser scanning confocal microscopy (Leica TCS SP8, Olympus FV1200 or ZEISS LSM 900) or upright microscopy (Leica DM6B) and processed using ImageJ or Fiji software (NIH).

### Stereological cell counts

The unbiased stereological cell count method was used to quantify dopaminergic neurons in the substantia nigra compacta (SNc), as previously described with minor modifications [42]. Briefly, brain slices were screened by upright microscopy (Leica DM6B). After that, TH positive cells in every 6th section encompassing the whole *substantia nigra* (SN) of each mouse were counted at × 40 magnification (7–8 sections per animal) using ImageJ software (NIH).

### Soluble and insoluble α-synuclein extraction

The striatum and substantia nigra (SN) tissues were isolated from mice brains and homogenized in 1% Triton X-100-containing extraction buffer supplemented with a cocktail of protease inhibitors [43]. The homogenate was centrifuged at 100,000 × g for 30 min at 4 °C using a Beckman Ultracentrifuge (Optima MAX-XP). After centrifugation, the supernatant obtained was the Triton-soluble fraction of the tissue. The insoluble pellet was resuspended in an extraction buffer containing 2% SDS and supplemented with protease inhibitor cocktails. Further, the lysate was centrifuged at 100,000 × g for 30 min at 20 °C after which the supernatant was labeled as the SDS-soluble fraction. Protein concentration of each sample was determined by the bicinchoninic acid (BCA) assay and adjusted to isoconcentration, aliquoted and stored at − 80 °C until use.

### Protein extraction of cell culture supernatants

After treatment, pooled supernatants were obtained and centrifuged at 1,000 × g for 5 min to remove cell debris [44]. To concentrate the samples via precipitation, 500 μL methanol and 125 μL chloroform were added to 500 μL supernatants and vigorously vortexed for at least 30 s. After 5 min of centrifugation at 13,000 × *g* and at 4 °C, the upper aqueous phase was carefully removed, 500 μL ice-cold methanol added to the remaining liquid, vigorously vortexed to break the protein layer into small white flakes after which repeated centrifugation was done for 5 min at 13,000 × *g* and at 4 °C. Supernatants were removed and pellets dried for 5–10 min at 55 °C. Then, pellets were reconstituted in 20 μL 2X loading buffer and denatured at 95 °C for 5 min.

### Immunoblotting (IB) analysis

Protein extracts from cell lysates, culture supernatants and mouse brain tissues were denatured in a loading buffer at 95 °C for 5 min and subjected to IB analysis. Protein samples were resolved and separated on SDS-PAGE accompanied by a protein ladder (Thermo Fisher Scientific cat# 26616 or #26619), and transferred to 0.2 μm polyvinylidene fluoride membranes (Merck Millipore). After blocking with 5% skim milk or 5% bovine serum albumin (BSA) in 0.1% Tween-20/Tris-buffered saline (TBS-T) for 1 h at RT, membranes were incubated overnight at 4 °C in the presence of primary antibodies (1:1000) under mild shaking. Then, membranes were washed thrice using TBS-T, incubated in the presence of horseradish peroxidase (HRP)-conjugated secondary antibodies (1:1000) for 1 h at RT under agitation, and washed thrice using TBS-T again. Finally, protein bands were visualized by chemiluminescence detection and imaged using a densitometer (Bio-Rad Imaging System, Hercules, CA). Band densities were quantified by ImageJ and normalized to GAPDH.

### Statistical analysis

GraphPad Prism 8.0 was used for data analyses. Data are expressed as mean ± SEM for at least *n* = 3. Comparisons of means among groups was performed by one-way or two-way ANOVA followed by Tukey’s multiple comparison tests. Statistical significances are indicated as *p < 0.05; **p < 0.01; ***p < 0.001; ****p < 0.0001; ^#^*p* < 0.05; ^##^*p* < 0.01; ^###^*p* < 0.001; ^####^*p* < 0.0001.

## Results

### Direct relationship between α-synuclein pathology and ASC expression levels

**Fig. 1:**
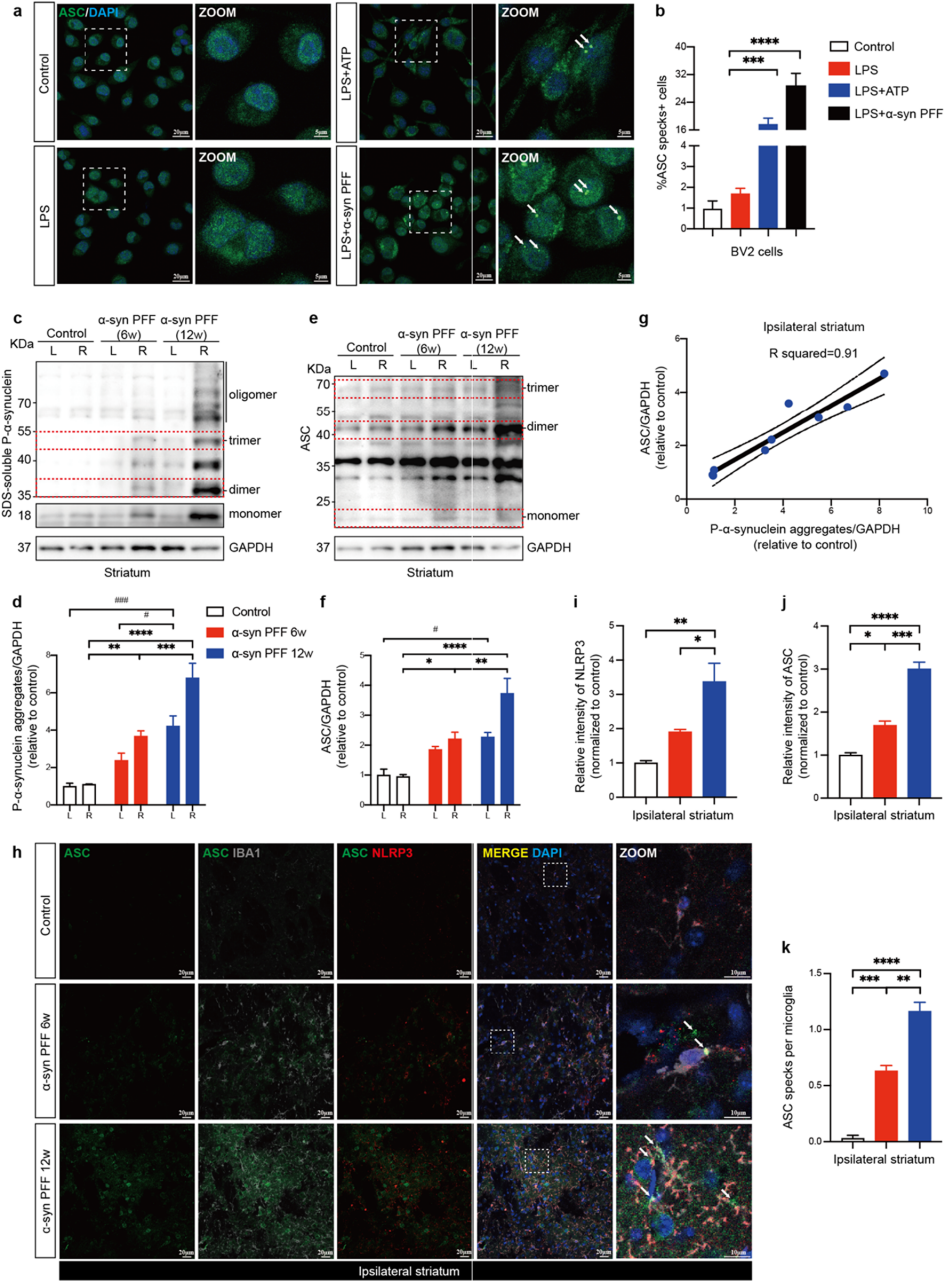
ASC specks formation upon PFFs stimulation correlated with progression of α-synuclein pathology. **a** Representative immunofluorescence staining of ASC (green) in BV2 cells stimulated with 1ug/ml LPS for 4 h followed by 5 mM ATP for 0.5 h or 2 μM human A53T mutant α-synuclein PFFs for 16 h. ATP was used as the positive control. DAPI represents the nuclear signal (blue). The white dotted boxes in images are magnified on the right. White arrows indicate ASC specks. **b** Quantification of the percentages of ASC specks-positive BV2 cells. **c**–**f** Immunoblot (IB) analysis and quantification of phosphate-α-synuclein aggregates and ASC levels in bilateral striatum of controls and human A53T mutant α-synuclein PFFs-induced PD mice at six- and twelve-weeks post-surgery (*n* = 3). The red dotted boxes in images indicate monomeric, dimeric or trimeric proteins. **g** A simple linear regression was performed on relative levels of ASC and phosphate-α-synuclein aggregates, and R squared was calculated (*n* = 9). **h** Representative immunofluorescence images of ASC (green), NLRP3 (red) and Iba1 (grey) in the ipsilateral striatum of indicated mice brains (*n* = 3). DAPI represents the nuclear signal (blue). The white dotted boxes in images are magnified on the right. White arrows indicate ASC specks. **i** Quantification of relative fluorescent intensities of NLRP3. (**j**) Quantification of relative fluorescent intensities of ASC. **k** Quantification of the average numbers of ASC specks per microglia in the indicated mice ipsilateral striatum. Data are presented as mean ± SEM for at least *n* = 3 and are analyzed by one-way or two-way ANOVA followed by Tukey’s post hoc test for multiple comparisons. Significance levels are indicated as: *p < 0.05, **p < 0.01, ***p < 0.001, ****p < 0.0001; ^#^*p* < 0.05; ^###^*p* < 0.001. Scale bars are as indicated. *L* left, *R* right, *LPS* lipopolysaccharide, *ATP* adenosine triphosphate

Fibrillar a-synuclein drives NLRP3 inflammasome activation and extracellular ASC specks release in primed microglia [9]. In our study, we reproduced this finding in lipopolysaccharide (LPS) primed BV2 cells using human A53T mutant α-synuclein PFFs instead of wildtype (WT) PFFs (Fig. 1a). We found a significant development of ASC specks in primed BV2 cells after 16 h of PFFs treatment, which was equivalent to that obtained after 0.5 h of activation using adenosine triphosphate (ATP) as the positive control (Fig. 1b). These findings indicated that A53T mutant α-synuclein PFFs could mediate a robust formation of the ASC specks in microglia. To further investigate the correlation between fibrillar α-synuclein stimulation and ASC expression levels in vivo, a mouse model of PD was induced by unilateral intrastriatal injection of sonicated human A53T mutant α-synuclein PFFs (Additional file 1: Fig. S1). Six- or twelve-weeks post-treatment, α-synuclein pathology expanded with disease progression, particularly in the striatum, substantia nigra (SN) and cortex of mice brains (Fig. 1c, d, Additional file 1: Fig. S2). Notably, ASC expression levels also increased with disease progression (Fig. 1e, f), which was positively and linearly correlated with the burden of phosphate-α-synuclein aggregates in the ipsilateral striatum (Fig. 1g). ASC specks were observed throughout the injection sites (Fig. 1h). In addition to the up-regulated NLRP3 and ASC intensities, the average numbers of ASC specks per microglia also increased in ipsilateral striatum as disease progressed (Fig. 1i–k), suggesting that ASC specks may be involved in the propagation of α-synuclein pathology [9, 45].

### ASC fibrils amplified NLRP3 inflammasome activation in PFFs-treated microglia

**Fig. 2:**
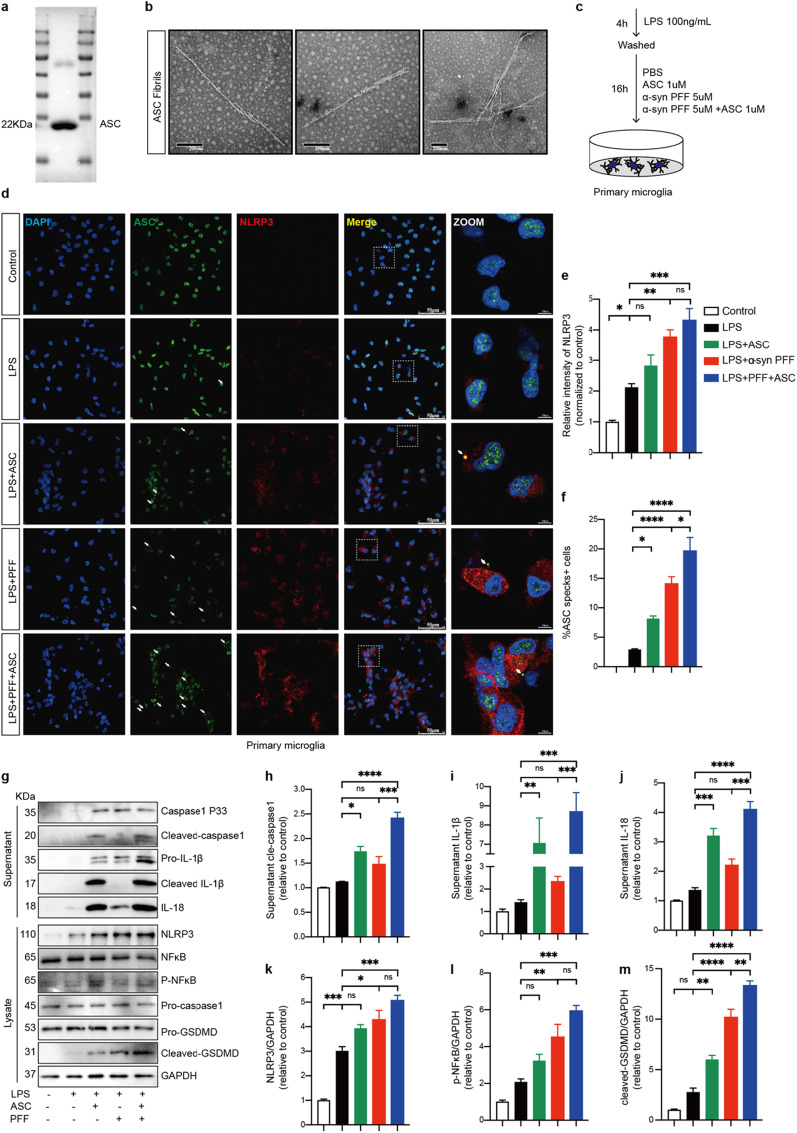
ASC fibrils amplified NLRP3 inflammasome activation in PFFs-treated microglia. **a** IB was probed for purified ASC proteins. 22 KDa: theoretical molecular weight of ASC monomer. **b** Representative transmission electron microscopy (TEM) images of ASC fibrils induced by incubation. **c** Schematic presentation of the following experimental setup. **d** Representative immunofluorescence staining of ASC (green) and NLRP3 (red) in primary microglia with indicated treatments. The white dotted boxes in images are magnified on the right. White arrows indicate ASC specks. **e** Quantification of relative fluorescent intensities of NLRP3. **f** Quantification of the percentages of ASC specks-positive primary microglia. **g**–**m** IB analysis and quantification of NLRP3 inflammasome signals in supernatants and cell lysates of PFFs or PBS-treated primed-primary microglia cells challenged with or without ASC specks. Data are shown as representative plots **g** and bands quantified by densitometric analysis (**h**–**m**). Data are presented as mean ± SEM for at least n = 3 and are analyzed by one-way ANOVA followed by Tukey’s post hoc test for multiple comparisons. Significance levels are indicated as: *p < 0.05, **p < 0.01, ***p < 0.001, ****p < 0.0001, *ns* no significant. Scale bars are as indicated

To validate the acquisition of recombinant ASC proteins, IB was performed using anti-ASC antibody. The determined molecular weight of main band was in good agreement with the theoretical molecular weight of 22 kDa (Fig. 2a). In TEM, ASC proteins were assembled and arranged into long, twisted helical filamentous structures after short time incubation at 37 °C in accordance with a previous study [46] (Fig. 2b). To assess the specific effect of ASC fibrils in primary microglia challenged by α‑synuclein PFFs, LPS-primed primary microglial cells were treated with ASC fibrils, PFFs with or without ASC fibrils, respectively (Fig. 2c). LPS priming of microglia induced elevated levels of NLRP3 but not formation of ASC specks (Fig. 2d–f). However, following PFFs or ASC fibrils treatment, a marked increase in the formation of ASC specks was observed in primed microglia. Interestingly, compared to LPS-primed microglia, an upregulation of NLRP3 intensity was only observed after PFFs treatment, but not after ASC fibrils treatment (Fig. 2d–f). Additionally, when primed cells were stimulated with PFFs and ASC fibrils together, the percentage of ASC specks-positive cells, but not NLRP3 levels, was significantly increased compared to PFFs treatment alone (Fig. 2d–f). These results were also confirmed using IB (Fig. 2g). In addition to assessing the expression levels of NLRP3 and phosphate-NF-κB, cleavage of GSDMD and caspase-1 and release of cytokines IL-1β, IL-18 into the extracellular space as consequences of NLRP3 inflammasome activation were evaluated. Compared with LPS-primed microglia, primed cells treated with PFFs alone induced increased levels of NLRP3, phosphate-NF-κB and cleaved GSDMD. However, for the levels of cleaved caspase-1, IL-1β, and IL-18, trends of increase were found but with no statistical significance. In contrast, when primed cells were treated with ASC fibrils alone, cleaved GSDMD, cleaved caspase-1, IL-1β, and IL-18 levels were remarkably upregulated, but NLRP3 and phosphate-NF-κB levels were not (Fig. 2h–m), indicating that ASC fibrils primarily amplified inflammasome activation (signal 2) rather than inflammasome priming (signal 1) in vitro. Only when primed cells were treated with PFFs and ASC fibrils together, NLRP3 inflammasome priming signaling (indicated as the expression levels of NLRP3 and phosphate-NF-κB) and activation signaling (indicated as the expression levels of cleaved caspase-1, cleaved GSDMD and cytokines) were significantly elevated (Fig. 2h–m). Collectively, these results demonstrated that ASC fibrils amplified NLRP3 inflammasome activation in PFFs-treated microglia.

### ASC specks enhanced NLRP3 inflammasome activation and reactive microgliosis in vivo

**Fig. 3:**
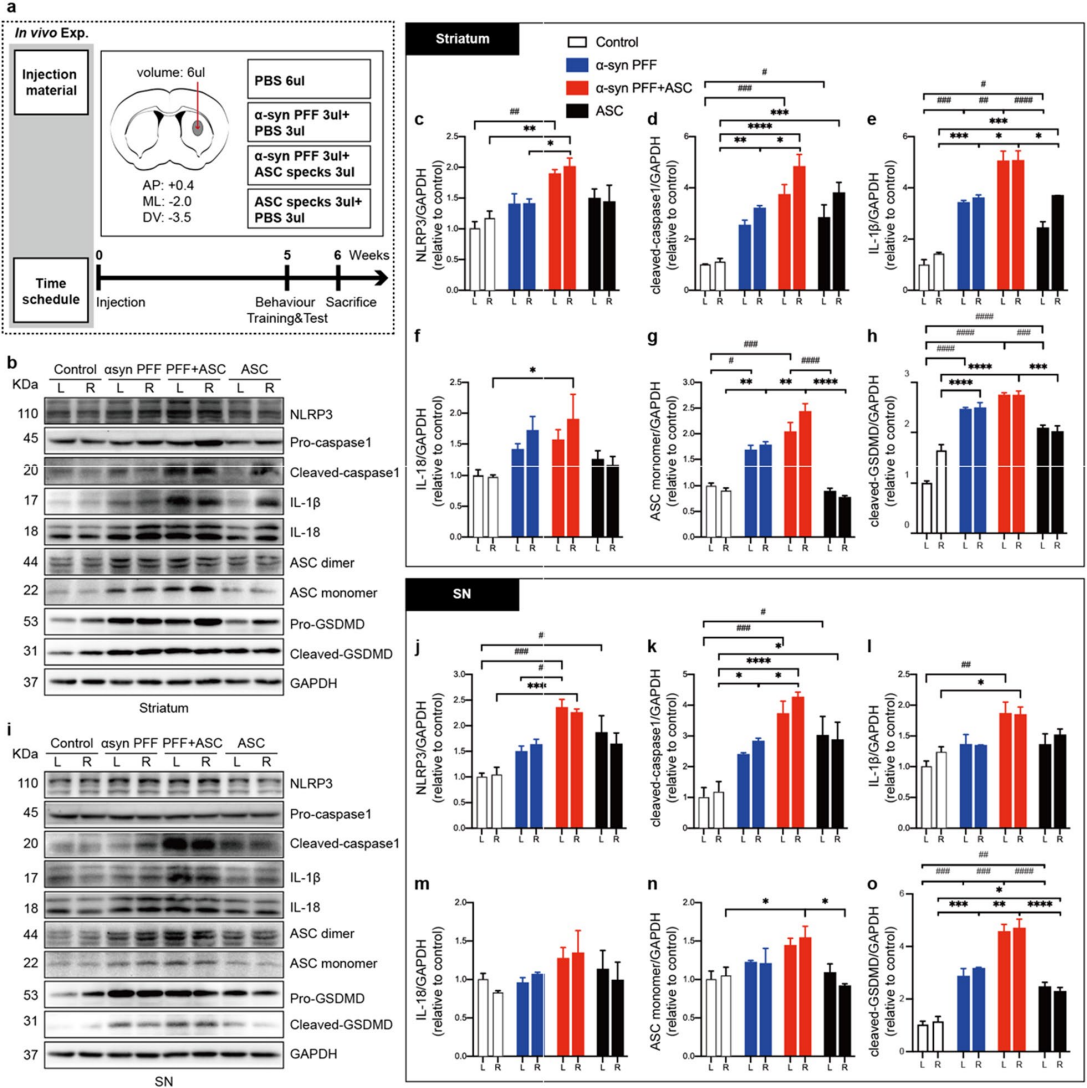
ASC specks augmented NLRP3 inflammasome activation in human A53T mutant α-synuclein PFFs-induced PD mice. **a** Diagram showing the experimental design and time course of treatment schedules in vivo. The number of mice in each group: PBS group, *n* = 11; PFFs treated group, *n* = 10; PFF + ASC co-injected group, *n* = 13; ASC specks treated group, *n* = 10. **b**–**h** IB analysis and quantification of NLRP3 inflammasome signals in bilateral striatum of indicated mice (*n* = 3 or 4). **i**–**o** IB analysis and quantification of NLRP3 inflammasome signals in bilateral SN of indicated mice (*n* = 3 or 4). Quantifications of NLRP3, cleaved-caspase 1, IL-1β, IL18, ASC monomer and cleaved GSDMD levels were performed by densitometric analysis normalized to GAPDH, relative to the contralateral striatum or SN regions of the control group. Values are presented as mean ± SEM and are analyzed by two-way ANOVA followed by Tukey’s post hoc test for multiple comparisons. Significance levels are: *p < 0.05, **p < 0.01, ***p < 0.001, ****p < 0.0001 (Right hemisphere of brain tissues); ^#^*p* < 0.05; ^##^*p* < 0.01; ^###^*p* < 0.001; ^####^*p* < 0.0001(Left hemisphere of brain tissues). *L* left, *R* right, *SN* substantia nigra

To further investigate the role of ASC specks on inflammasome activation in PD in vivo, ASC specks were co-injected into the ipsilateral striatum in a mouse model of PD induced by human A53T mutant α-synuclein PFFs and analyzed at 6 weeks post-surgery (Fig. 3a). Levels of NLRP3 inflammasome signals in the striatum and SN regions of indicated mice were evaluated using IB. Prolonged stimulation of PFFs in vivo induced upregulation of cleaved GSDMD, cleaved caspase 1, IL1β and ASC levels, while a similar but non-statistically significant trends were found for NLRP3 and IL-18 levels (Fig. 3b–h). However, the levels of NLRP3, as well as cleaved GSDMD, cleaved caspase-1, IL-1β, IL18 and ASC proteins were significantly increased in ipsilateral or bilateral striatum of mice co-injected with ASC specks and PFFs, indicating that ASC specks promoted both priming and activation of NLRP3 inflammasome in vivo (Fig. 3b–h). Similar alterations of NLRP3 inflammasome signals were observed in bilateral SN regions of indicated mice brains (Fig. 3i–o).

**Fig. 4:**
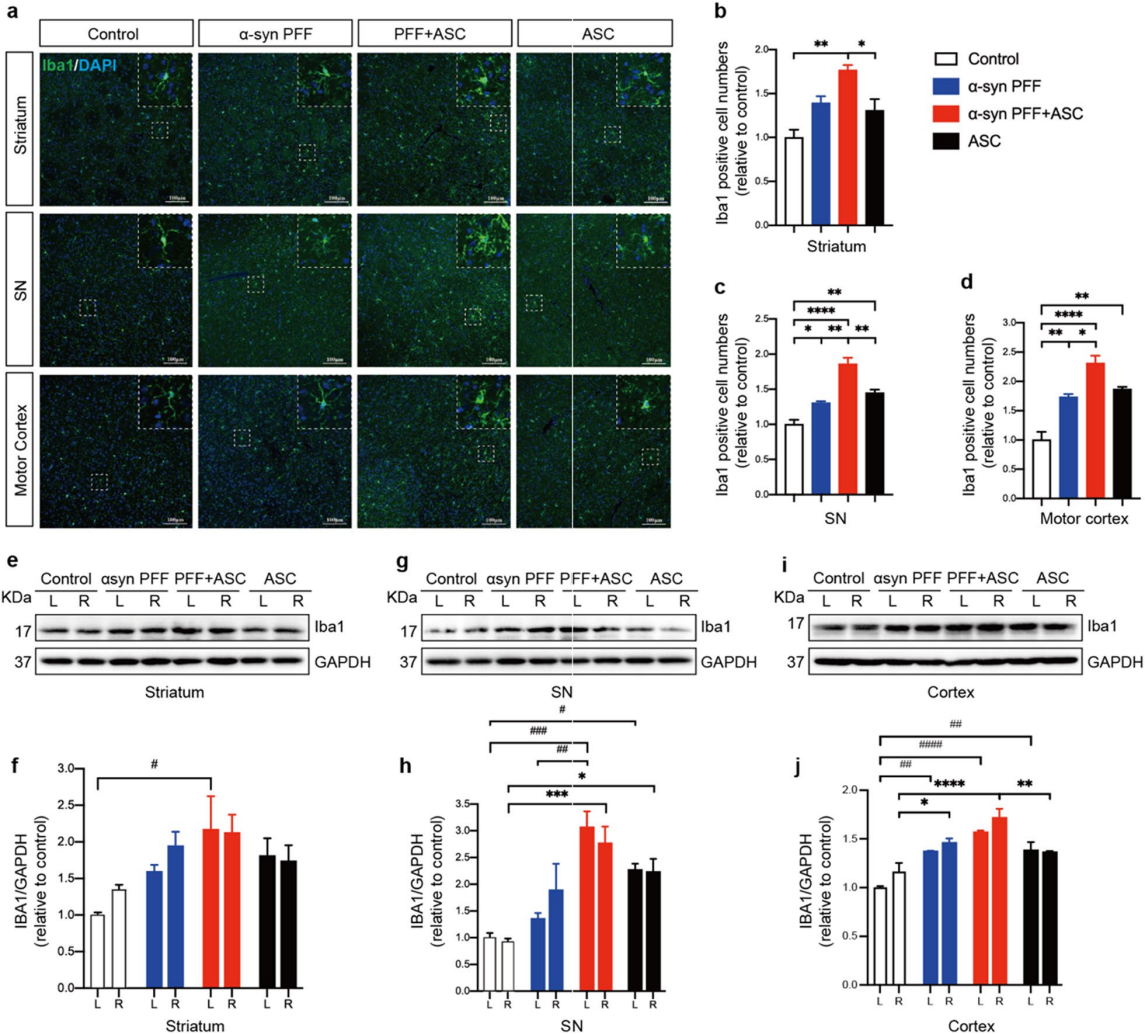
ASC specks enhanced reactive microgliosis. **a** Representative immunofluorescence staining of Iba1 (green) in ipsilateral striatum, SN and motor cortex of indicated mice brains. White dotted squares show the morphology of the microglia after magnification. **b**–**d** Quantification of the relative Iba1-positive cell numbers in ipsilateral striatum, SN and motor cortex of indicated mice brains (*n* = 3 or 4). **e**, **f** IB analysis and quantification of Iba1 in bilateral striatum (*n* = 3 or 4). **g**, **h** IB analysis and quantification of Iba1 in bilateral SN tissues (n = 3 or 4). **i**, **j** IB analysis and quantification of Iba1 in bilateral cortex (*n* = 3 or 4). Values are presented as mean ± SEM and are analyzed by one-way or two-way ANOVA followed by Tukey’s post hoc test for multiple comparisons. Significance levels are: *p < 0.05, **p < 0.01, ***p < 0.001, ****p < 0.0001 (Right hemisphere of brain tissues); ^#^*p* < 0.05; ^##^*p* < 0.01; ^###^*p* < 0.001; ^####^*p* < 0.0001 (Left hemisphere of brain tissues). Scale bar: 100 μm. *L* left, *R* right, *SN* substantia nigra

Given that NLRP3 inflammasome activation is mainly performed in the microglia, we stained for ionized calcium-binding adapter molecule 1 (Iba1) to compare changes in microglial numbers and morphologies in different groups to define microglial activities. Iba1, also known as allograft inflammatory factor 1 (AIF-1), is upregulated during the activation of microglia [47]. We observed that microglia in ASC specks and PFFs co-injected group markedly proliferated and exhibited an amoeba shape with a large soma and a small perimeter in ipsilateral striatum, SN and motor cortex (Fig. 4a–d). IB results from striatum, SN tissues and cortex also demonstrated the elevated levels of Iba1 protein in the co-injection group (Fig. 4e–j), indicating that ASC specks enhanced reactive microgliosis.

### ASC specks exacerbated α-synuclein pathology

**Fig. 5:**
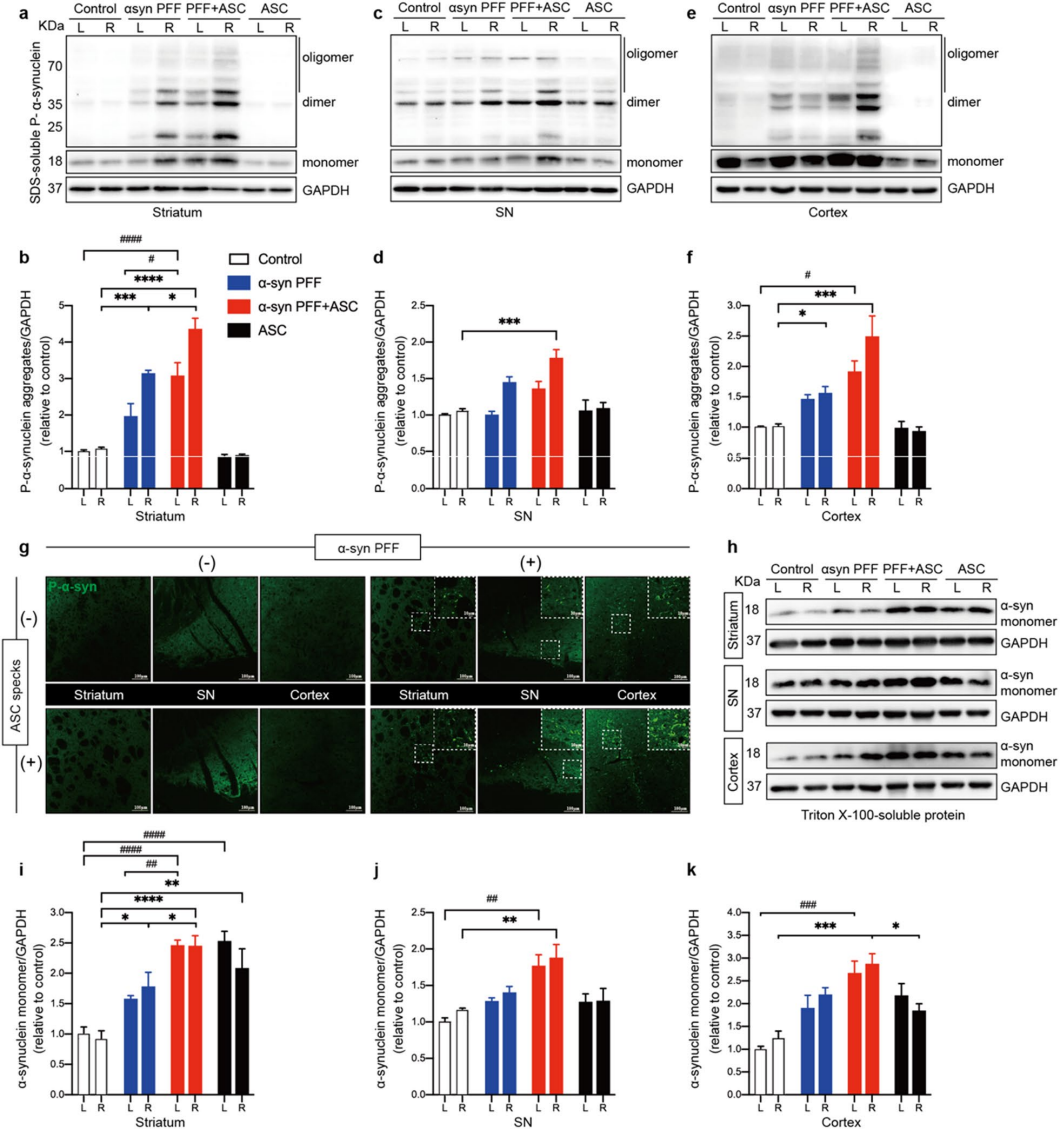
ASC specks exacerbated α-synuclein aggregation and propagation. **a**, **b** IB analysis and quantification of SDS-soluble phosphate-α-synuclein (P-α-syn) in bilateral striatum (*n* = 4 or 6). **c**, **d** IB analysis and quantification of SDS-soluble P-α-syn in bilateral SN tissues (*n* = 4 or 6). **e**, **f** IB analysis and quantification of SDS-soluble P-α-syn in cortex (*n* = 4 or 6). **g** Representative immunofluorescence staining of P-α-syn (green) in the ipsilateral striatum and SN regions of indicated mice. **h**–**k** IB analysis and quantification of triton-soluble α-synuclein monomers in the bilateral striatum, SN and cortex in indicated mice (*n* = 4 or 6). Data are presented as mean ± SEM and are analyzed by two-way ANOVA followed by Tukey’s post hoc test for multiple comparisons. Significance levels are: *p < 0.05, **p < 0.01, ***p < 0.001, ****p < 0.0001 (Right hemisphere of brain tissue); ^#^*p* < 0.05; ^##^*p* < 0.01; ^###^*p* < 0.001; ^####^*p* < 0.0001 (Left hemisphere of brain tissue). Scale bars are as indicated. *L* left, *R* right, *SN* substantia nigra

To determine whether ASC specks accelerate α-synuclein aggregation and spreading in vivo, we assessed the levels of insoluble phosphate-α-synuclein aggregates from indicated mice brains using a detergent-containing solution under ultracentrifugation. Intrastriatal injections of ASC specks in PFFs-induced PD mice substantially increased phosphate-α-synuclein aggregates in ipsilateral striatum, when compared to PFFs treatment alone (Fig. 5a, b). Although no significant differences of phosphate-α-synuclein aggregates in SN and cortical tissues were observed between the co-injection group and the PFFs treatment alone group, the mean levels of aggregates remained higher in the co-injection group (Fig. 5c–f). These findings were also confirmed by immunofluorescence of phosphate-α-synuclein deposits in ipsilateral striatum, SN and cortex of mice brains (Fig. 5g). In addition, we found that ASC specks co-injections enhanced levels of Triton X-100-soluble α-synuclein monomers in the brains of PD mice, in line with the results of phosphate-α-synuclein aggregates (Fig. 5h–k). Interestingly, levels of α-synuclein monomers were also increased in the brains of mice treated with ASC specks alone, especially in the bilateral striatum (Fig. 5h–k). Given that upregulation of α-synuclein levels following inflammatory activation may contribute to α-synuclein aggregation [48], these results further supported that ASC specks could exacerbate α-synuclein pathology. However, mice treated with ASC specks alone lacked α-synuclein pathology (Fig. 5a–g), implying that ASC specks can only accelerate α-synuclein accumulation but not initiate it.

### ASC specks accelerated dopaminergic neuron degeneration and dyskinesia

**Fig. 6:**
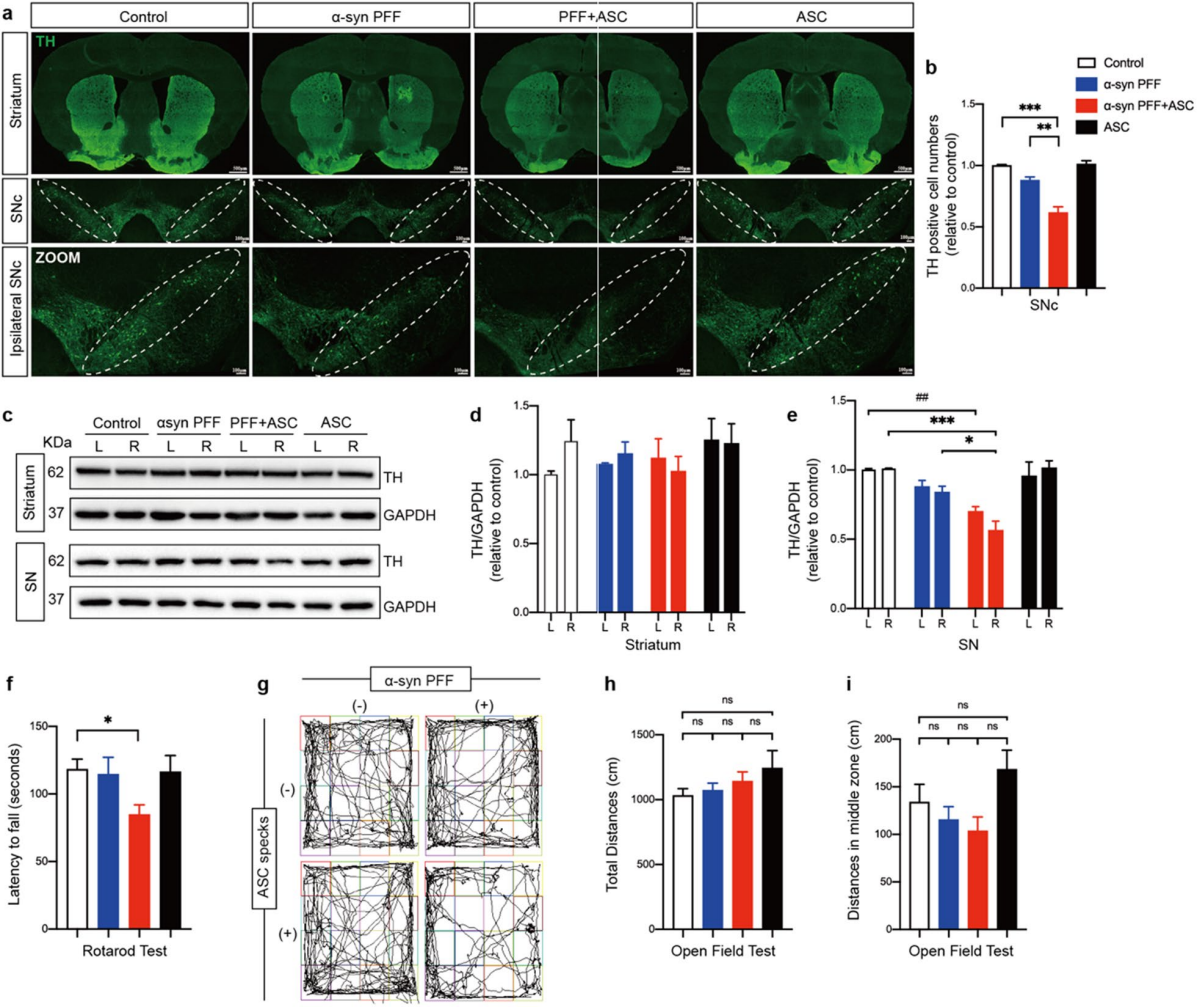
ASC specks accelerated dopaminergic neurons degeneration and motor deficits. **a** Representative immunofluorescence staining of tyrosine hydroxylase (TH) positive neurons (green) in the striatum and substantia nigra compacta (SNc) of indicated mice brains. White dotted circles indicate SNc regions. Ipsilateral SNc regions in images are magnified below. **b** Quantification of percentages of relative TH positive cells in SNc (*n* = 3 or 4). **c**–**e** IB was performed to determine TH levels in bilateral striatum and SN of indicated mice brains (*n* = 3 or 4). Data are shown as representative plots **c** and bands quantified by densitometric analysis (**d**, **e**). **f** Latency to falling during the rotarod test was recorded and analyzed for each mouse. **g** Representative images of general locomotor activities and exploratory behaviors of indicated mice in the open field test. **h**, **i** Total distance traveled and distance traveled in middle zone were recorded and analyzed for each mouse. Data are presented as mean ± SEM and are analyzed by one-way or two-way ANOVA followed by Tukey’s post hoc test for multiple comparisons. Significance levels are denoted as: *p < 0.05, **p < 0.01, ***p < 0.001; ^##^*p* < 0.01; *ns* no significant. Scale bars are as indicated. *L* left, *R* right, *SNc* substantia nigra compacta, *SN* substantia nigra

To characterize the overall effects of ASC specks on neuropathology and associated behavioral functions in vivo, we assessed the survival of dopaminergic neurons and motor behaviors in the indicated mice. Tyrosine hydroxylase (TH), a rate-limiting enzyme responsible for biosynthesis of dopamine, deficiency of which reflects the loss of dopaminergic neurons and the progression of PD [49]. Unbiased stereological counts of tyrosine hydroxylase (TH) positive neurons in the substantia nigra compacta (SNc) indicated that co-injection of ASC specks resulted in a ~ 25% reduction in the number of TH positive neurons compared to PFFs treatment alone (Fig. 6a, b). This neuro-damaging effect of ASC specks was further supported by quantification of TH expression levels using IB in the bilateral SN, although this effect was absent in the bilateral striatum (Fig. 6c–e). Furthermore, compared to controls, mice treated with PFFs or ASC specks alone showed no significant difference in motor behaviors including open field and rotarod tests, while mice co-injected with ASC specks and PFFs exhibited significant deteriorations on the rotarod test (Fig. 6f–i). These findings indicated that co-injections of ASC specks accelerated neurodegeneration and dyskinesia in PD.

### ASC knockdown suppressed NLRP3 inflammasome activation and α-synuclein accumulation

**Fig. 7:**
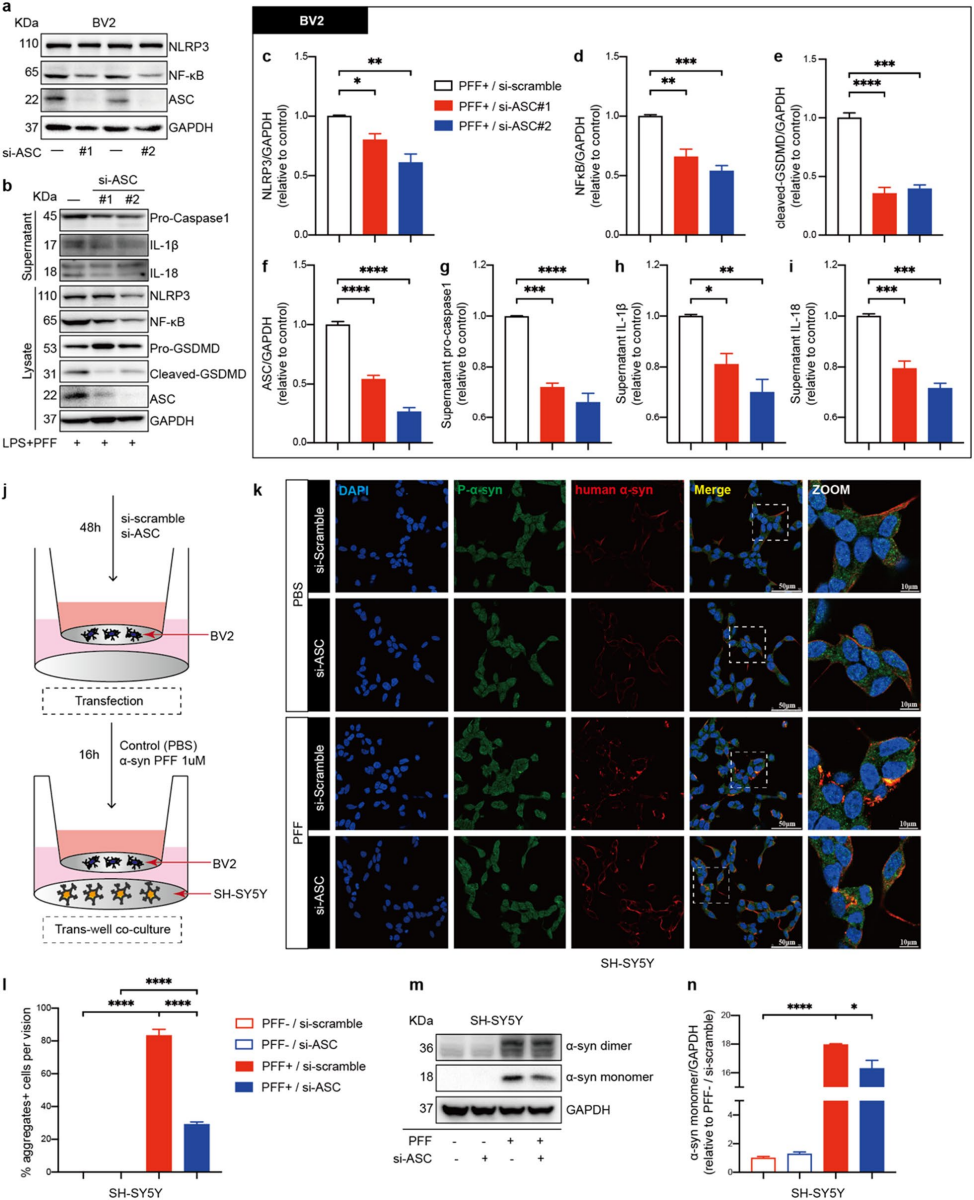
ASC knockdown suppressed PFFs-induced NLRP3 inflammasome activation in BV2 cells and upregulation of α-synuclein in SH-SY5Y cells. **a** IB analysis of NLRP3, NF-κB, ASC in mouse BV2 cells transfected with either si-scramble RNA or si-ASC RNA for 48 h. **b**–**i** IB analysis and quantification of NLRP3 inflammasome signals in BV2 cells challenged with 500 ng/ml LPS priming for 4 h followed by 1 μM PFFs stimulation for 16 h after transfection. **j** Schematic drawing of the BV2 cells and SH-SY5Y cells co-culture setup used in this study. Si-scramble RNA or si-ASC RNA transfected BV2 cells are treated with PBS or 1 μM PFFs and co-cultured with SH-SY5Y cells for 16 h. **k** Representative immunofluorescence images of P-α-syn (green) and human α-synuclein (red) in SH-SY5Y cells co-cultured with indicated-treated BV2 cells. The white dotted boxes in images are magnified on the right. **l** Quantification of the percentages of α-synuclein aggregates-positive cells per vision in SH-SY5Y cells. **m**, **n** IB analysis and quantification of α-synuclein in co-cultured SH-SY5Y cells. Data are obtained from at least three independent experiments. Data are presented as mean ± SEM and are analyzed by one-way ANOVA followed by Tukey’s post hoc test for multiple comparisons. Significance levels are: *p < 0.05, **p < 0.01, ***p < 0.001, ****p < 0.0001. Scale bars are as indicated

To further verify the pathogenic role of endogenous ASC in vitro, expression of ASC protein in BV2 cells was downregulated using si-ASC/PYCARD RNA (Fig. 7a). Knockdown of ASC significantly suppressed NLRP3 inflammasome activation when LPS-primed BV2 cells were treated with PFFs (Fig. 7b). Specifically, ASC knockdown suppressed NLRP3 and NF-κB levels and inhibited the cleavage of GSDMD, release of caspase-1, IL-1 β and IL-18, indicating that endogenous ASC regulated priming and activation of NLRP3 inflammasomes triggered by PFFs (Fig. 7c–i). To determine the effects of ASC knockdown in BV2 cells on neuronal cells, BV2 cells and SH-SY5Y cells were co-cultured in a trans-well system (Fig. 7j). When the co-culture systems were stimulated with PFFs, significant reduction of α-synuclein levels was observed in SH-SY5Y cells co-cultured with si-ASC RNA-transfected BV2 cells, compared to those co-cultured with si-scramble RNA-transfected BV2 cells (Fig. 7k–n). Specifically, α-synuclein in SH-SY5Y cells aggregated and colocalized with phosphate-α-synuclein when co-cultured cells were treated with PFFs, whereas ASC knockdown in BV2 cells partially reversed α-synuclein aggregation and colocalized plaque formation (Fig. 7k, l). These findings were supported by IB analyses, showing that when ASC was downregulated in BV2 cells, the expression levels of α-synuclein monomers were decreased in SH-SY5Y cells (Fig. 7m, n). These results indicated that ASC knockdown has protective effects on both microglial and neuronal cells.

Experiments targeting ASC specks using anti-ASC specks antibodies in PFFs-induced PD mice were performed to elucidate on the pathogenic effects of ASC specks in vivo (Additional file 1: Fig. S3a). The findings were comparable for almost all parameters in mice treated with PFFs alone and in mice treated with PFFs with either anti-IgG isotype antibodies or anti-ASC specks antibodies (Additional file 1: Figs. S3 and S4), which possibly related to the limited number of group samples, time and dosage of reagent injections or the controversial therapeutic efficacy of the antibody itself.

## Discussion

**Fig. 8:**
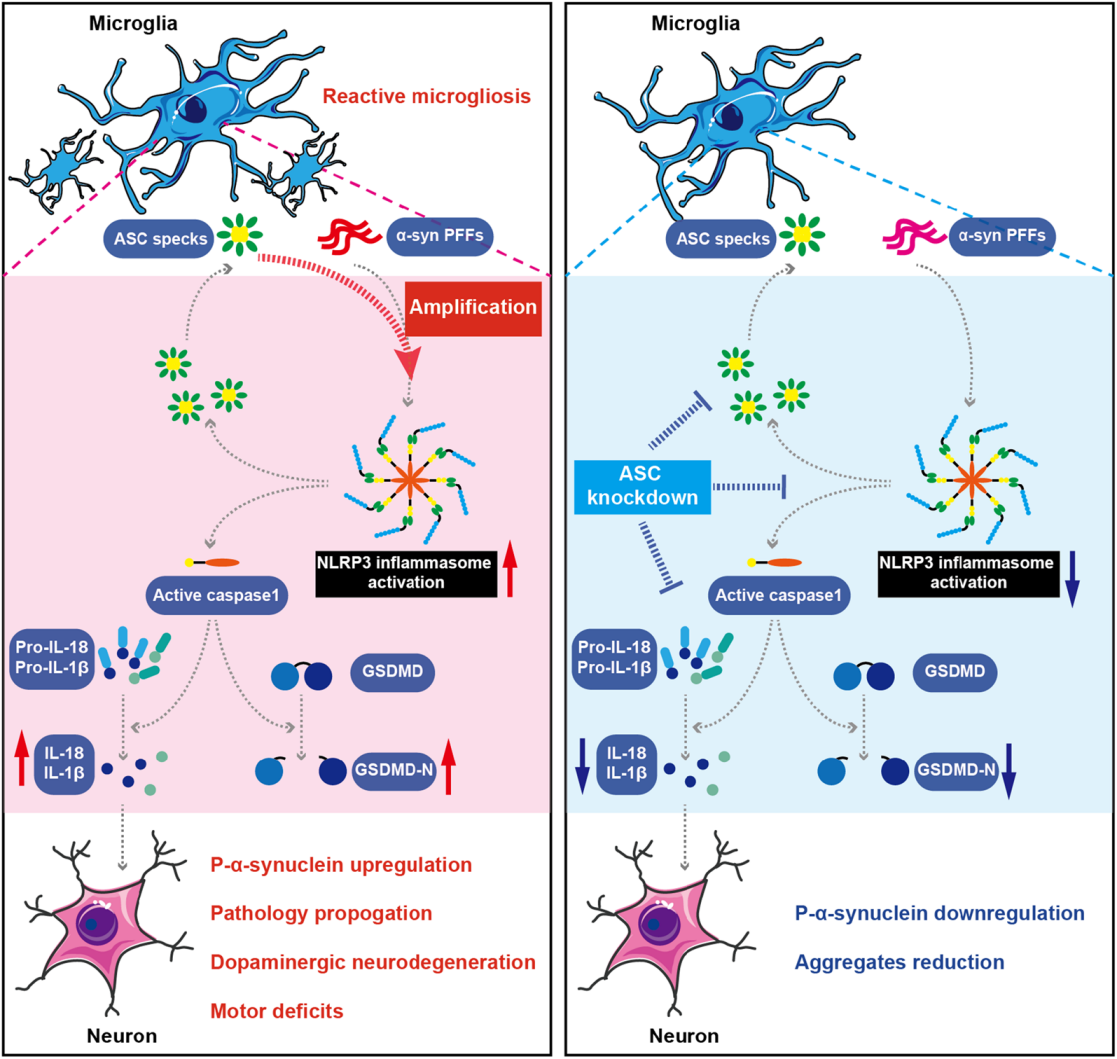
ASC specks exacerbate neuronal α‑synuclein pathology via amplifying microglial NLRP3 inflammasome activities. The NLRP3 inflammasome was assembled and activated in microglia under α-synuclein PFFs stimulation. Upon the assembly of ASC with NLRP3, pro-caspase-1 was recruited, which mediated cleavage of cytokines and GSDMD, accompanied with ASC specks formation and release. ASC specks amplified NLRP3 inflammasome activation induced by α-synuclein PFFs in a vicious positive feedback manner, which significantly promoted reactive microgliosis and neuronal α-synuclein accumulation. These effects contributed to propagation of α-synuclein pathology, degeneration of dopaminergic neurons and motor deficits. Knockdown of endogenous ASC significantly suppressed microglial NLRP3 inflammasome activation and neuronal α‑synuclein aggregation under the challenge of PFFs, indicating that targeting ASC is a potentially therapeutic approach for PD

In this work, we (i) identified human A53T mutant α-synuclein PFF as an effective activator of NLRP3 inflammasome. We (ii) established a direct relationship between levels of ASC protein and misfolded α‑synuclein aggregates in vivo. We (iii) determined that ASC specks amplified NLRP3 inflammasome activation and reactive microgliosis, exacerbating α‑synuclein pathology, dopaminergic neurodegeneration and motor deficits. Our findings thus provide novel insights into the mechanisms of PFFs-induced inflammasome activation and its contribution to propagation of α-synuclein pathology. Importantly, (iv) the ASC protein presents as a promising target, as endogenous knockdown of ASC in microglia protected both microglial and neuronal cells (Fig. 8).

Numerous neurodegenerative diseases share commonalties in their pathogenesis through activation of the NLRP3 inflammasome [50, 51]. Recent studies have implicated that NLRP3 inflammasome and the ASC specks play important roles in the propagation and spreading of neuroinflammation and misfolded protein aggregation in neurodegenerative diseases [4, 52]. Basic research on the underlying mechanisms of NLRP3 inflammasome activation in PD has received extensive attention [53–56]. However, the potential role of ASC specks in PD progression has not been conclusively determined. Franklin et al. were the first to demonstrate that ASC protein has extracellular and ‘prionoid’ activities [57]. ASC specks released from inflammasomes-activated cells remain active in the extracellular space and behave like exogenous danger signals. They can be taken up by recipient cells and aggregate cytosolic soluble ASC from recipient cells via a prion-like manner [57]. The prion-like structure of ASC specks is resistant to proteolytic degradation, serving as amplification mechanisms for inflammasome signaling and leading to the toxic, chronic progressive inflammatory state [19, 58]. Importantly, these extracellular ASC specks have been commonly seen in models of neurodegenerative diseases [4]. Studies on AD have shown that ASC expressions increase with age in APP/PS1, but not in wild-type mice [59, 60]. Our results also demonstrated that ASC levels positively correlate with α‑synuclein pathology as the disease progresses, accompanied by an increase in the number of ASC specks. This finding was consistent with what we observed when quantifying the levels of these two proteins in plasma exosomes from PD patients [61], suggesting that ASC protein may be involved in the propagation of inflammasome-involved pathology triggered by PFFs.

Therefore, our study here investigated the specific role of ASC specks in inflammasome activation triggered by α‑synuclein PFFs in vitro and in vivo. It has been documented that fibrillar α‑synuclein induces a robust NLRP3 inflammasome activation with a delayed release of caspase-1 and IL-1β until 24 h [9]. Consistently, in our study, there was a significant increase in NLRP3 and phosphate-NF-κB levels in primed microglia after 16 h of PFFs treatment, while a similar but non-statistically significant trends were found for cleaved caspase-1 and IL-1β. However, PFFs and ASC specks co-treatment significantly enhanced the cleavage of caspase-1 and secretion of IL-1β as well as IL-18 but not levels of NLRP3 or phosphate-NF-κB, indicating that ASC specks primarily amplified NLRP3 inflammasome activation rather than priming under LPS-primed microglia conditions in vitro*.* Notably, the contribution of ASC specks on inflammasome activation in vivo were slightly different*.* Compared to mice treated with PFFs alone, mice co-injected with ASC specks and PFFs exhibited significantly up-regulated priming and activation reporters of NLRP3 inflammasomes, accompanied by reactive microgliosis.

Furthermore, to characterize the overall effects of ASC specks on disease pathological progression in vivo, we assessed the α‑synuclein pathology, survival of dopaminergic neurons and motor behaviors in the indicated mice. Notably, the time points at which pathological inclusions and motor deficits appear in PD models significantly vary depending on types, doses and sites of fibril injection [62–64]. After six weeks treatment, substantial increases in α-synuclein and phosphate-α-synuclein levels were observed in ASC specks co-injected PD mice, indicating that ASC specks accelerate PD pathological progression. Interestingly, levels of α-synuclein monomers were also elevated in the brains of mice treated with ASC specks alone. Nonetheless, no pathological α-synuclein deposition was observed in these mice, suggesting that ASC specks primarily accelerate α-synuclein pathology rather than initiate it. No significant differences in neuronal survival and motor functions were observed in our PFFs-induced PD mice models when compared to controls, whereas mice co-injected with ASC specks exhibited significant deterioration in dopaminergic neuron survival and rotarod tests.

Interestingly, compared to ASC knockout, genetic deficiency in NLRP3 only partially impaired IL-1β responses to ASC specks in BMDMs [57], suggesting the significance of ASC protein rather than NLRP3 protein in inflammatory regulation. Therefore, we evaluated the neuroprotective effect of ASC inhibition by co-culturing SH-SY5Y cells with endogenous ASC-knockdown BV2 cells. Knockdown of ASC significantly inhibited NLRP3 inflammasome activation, GSDMD cleavage and inflammatory cytokines release when BV2 cells were stimulated by PFFs. Additionally, ASC knockdown suppressed neuronal α-synuclein expression and aggregation, indicating that ASC inhibition has protective effects on both microglia and neurons. Notably, previous studies have demonstrated that GSDMD is required for IL-1β secretion in inflammasome responses, and that IL-1β release may contribute to neuronal dysfunction and pathology [17, 65]. Further studies using GSDMD knockout mice are required to address the mechanisms underlying the effects of ASC specks on microglial and neuronal interactions. Together, these in vitro and in vivo findings demonstrated that ASC protein contribute to α-synuclein pathology by amplifying activation of the microglial NLRP3 inflammasome in PD.

To further verify the therapeutic significance of ASC specks inhibition in vivo, targeting of ASC specks via antibody co-incubation was performed in our PD mice models according to a previous study in AD [59]. However, no differences were observed in PFFs-induced PD mice receiving either isotype-specific IgG or ASC specks-specific IgG antibodies. Although protective effects of anti-ASC specks antibodies in AD, multiple sclerosis and spinal cord injury have been reported [59, 66, 67], the efficacy of anti-ASC antibody therapy has not been fully established. In a peritonitis model induced by intraperitoneal injection of silica crystals, anti-ASC treatment opsonized ASC specks and increased inflammation, potentially exacerbating the effects of extracellular ASC specks. Therefore, there is a need for careful design and selection of antibodies against ASC for future therapeutic applications [57]. Furthermore, ASC prion-like polymerization is an evolutionarily conserved mechanism of signal transduction in innate immune responses, indicating underlying evolutionary benefits [58]. Given differences in experimental time, injection dose and controversial therapeutic efficacies of the anti-ASC antibody, studies should be conducted to elucidate on how to better modulate ASC activity to improve the value of anti-ASC antibodies in regulating inflammation, and to identify the significance of ASC as a promising treatment target for PD.

## Conclusions

There is a clear association between ASC specks assembly, NLRP3 inflammasome activation and PD pathological progression, especially with regards to α‑synuclein accumulation. In PD, ASC specks significantly amplified the activation of NLRP3 inflammasome and reactive microgliosis, promoting α‑synuclein pathology in a vicious positive-feedback manner; ASC specks also contributed to earlier occurrence of dopaminergic neurodegeneration and dyskinesia; Knockdown of endogenous ASC markedly suppressed microglial inflammasome activation and neuronal α‑synuclein aggregation under the challenge of PFFs. These findings imply that targeting ASC is a promising therapeutic approach for PD.

## Supplementary Information

**Additional file 1: Fig. S1** Preparation of sonicated α-synuclein PFFs for stereotaxic injections. Related to Fig. 1.** a**, **b** Representative TEM images of recombinant human A53T mutant α-synuclein PFFs before and after sonication. Scale bar: 500 nm. **Fig. S2** Propagation of α-synuclein pathology in the mice brains as PD progresses. Related to Fig. 1.** a**–**c** Representative immunofluorescence images of P-α-syn (green) in several brain slices of control mice and PFFs-induced PD mice after six- and twelve- weeks treatment. The colored boxes in images are magnified on the right. **Fig. S3** No significant protective effects of anti-ASC antibody were found in PFFs-treated PD mice. Related to Fig. 7. **a** Time schedules of the in vivo experiment in this study. The number of mice in each group: control group, *n* = 9; PFF group, *n* = 9; PFF + anti-IgG group, *n* = 12; PFF + anti-ASC group, *n* = 10. **b** Representative images of the tracks of indicated mice in open field test. **c**, **d** The total distance traveled and distance traveled in middle zone were recorded and analyzed for each mouse. **e** The latency to falling during the rotarod test was recorded and analyzed for each mouse. **f** Representative immunofluorescence staining of TH (green) neurons in the striatum and SNc of mice brains (*n* = 3–6). White dotted circles indicate the SNc regions. **g** IB analysis and quantification of TH in bilateral striatum and SN of indicated mice (*n* = 3). **h** Representative immunofluorescence staining of Iba1 (green) in the ipsilateral striatum, SN and motor cortex of mice brains (*n* = 3–6). White dotted squares show the morphology of microglia after magnification. **i** IB analysis of Iba1 in bilateral striatum and SN of indicated mice (*n* = 3). Values are presented as mean ± SEM and are analyzed by one-way or two-way ANOVA followed by Tukey’s post hoc test for multiple comparisons. Levels of significance are: *p < 0.05; ns, no significant. Scale bars are as indicated. L, left; R, right; SNc, substantia nigra compacta; SN, substantia nigra. **Fig. S4** No differences in α-synuclein pathology and NLRP3 inflammasome activation were observed in brain tissues of anti-ASC antibody treated or anti-isotype treated PD mice. Related to Fig. 7. **a** Representative immunofluorescence staining of p-α-syn (green) in ipsilateral striatum, SN and cortex of indicated mice (*n* = 3–6). **b–d** IB analysis and quantification of SDS-soluble p-α-syn in bilateral striatum and SN tissues (*n* = 3). **e**, **f** IB analysis and quantification of NLRP3 inflammasome signals in bilateral striatum and SN of indicated mice (*n* = 3). Values are presented as mean ± SEM and are analyzed by two-way ANOVA followed by Tukey’s post hoc test for multiple comparisons. Levels of significance are indicated as follows: *p < 0.05; ns, no significant. Scale bar: 50 μm. *L* left, *R* right, *SN* substantia nigra.

## Data Availability

All data generated or analyzed during this study are included in this published article and its supplementary information files.

## Abbreviations

AD: Alzheimer’s disease
AIF-1: Allograft inflammatory factor 1
AP: Anterior–posterior
ASC: Apoptosis-associated speck-like protein containing a CARD
ATP: Adenosine triphosphate
BCA: Bicinchoninic acid
caspases: Cysteinyl aspartate-specific proteinases
CSF: Colony-stimulating factor
DAMPs: Damage-associated molecular patterns
DAPI: 4,6-Diamidino-2-phenylindole
DMEM: Dulbecco’s modified Eagle’s medium
GSDMD: Gasdermin-D
DV: Dorsal–ventral
FBS: Fetal bovine serum
HRP: Horseradish peroxidase
IB: Immunoblotting
Iba1: Ionized calcium-binding adapter molecule 1
IPTG: Isopropyl b-D-1-thiogalactopyranoside
LPS: Lipopolysaccharide
ML: Medial–lateral
NLRP3: NLR family pyrin domain-containing 3
P-α-syn: Phosphate-α-synuclein
PAMPs: Pathogen-associated molecular patterns
PBMCs: Peripheral blood mononuclear cells
PBS: Phosphate buffered saline
PD: Parkinson’s disease
PDL: Poly-D-lysine
PFA: Paraformaldehyde
PFFs: Preformed fibrils
ROS: Reactive oxygen species
SN: Substantia nigra
SNc: Substantia nigra compacta
TBS-T: Tween-20/Tris-buffered saline
TEM: Transmission electron microscopy
TH: Tyrosine hydroxylase
ThT: Thioflavin T
