# Supplementary material for: ASC specks exacerbate α‑synuclein pathology via amplifying NLRP3 inflammasome activities
Source: J Neuroinflammation. 2023 Feb 5;20:26. doi: 10.1186/s12974-023-02709-w (PMC9899382; doi:10.1186/s12974-023-02709-w)
Supplement: Supplementary file 1 — Additional file 1: Fig. S1 Preparation of sonicated α-synuclein PFFs for stereotaxic injections. Related to Fig. 1. a, b Representative TEM images of recombinant human A53T mutant α-synuclein PFFs before and after sonication. Scale bar: 500 nm. Fig. S2 Propagation of α-synuclein pathology in the mice brains as PD progresses. Related to Fig. 1. a–c Representative immunofluorescence images of P-α-syn (green) in several brain slices of control mice and PFFs-induced PD mice after six- and twelve- weeks treatment. The colored boxes in images are magnified on the right. Fig. S3 No significant protective effects of anti-ASC antibody were found in PFFs-treated PD mice. Related to Fig. 7. a Time schedules of the in vivo experiment in this study. The number of mice in each group: control group, n = 9; PFF group, n = 9; PFF + anti-IgG group, n = 12; PFF + anti-ASC group, n = 10. b Representative images of the tracks of indicated mice in open field test. c, d The total distance traveled and distance traveled in middle zone were recorded and analyzed for each mouse. e The latency to falling during the rotarod test was recorded and analyzed for each mouse. f Representative immunofluorescence staining of TH (green) neurons in the striatum and SNc of mice brains (n = 3–6). White dotted circles indicate the SNc regions. g IB analysis and quantification of TH in bilateral striatum and SN of indicated mice (n = 3). h Representative immunofluorescence staining of Iba1 (green) in the ipsilateral striatum, SN and motor cortex of mice brains (n = 3–6). White dotted squares show the morphology of microglia after magnification. i IB analysis of Iba1 in bilateral striatum and SN of indicated mice (n = 3). Values are presented as mean ± SEM and are analyzed by one-way or two-way ANOVA followed by Tukey’s post hoc test for multiple comparisons. Levels of significance are: *p < 0.05; ns, no significant. Scale bars are as indicated. L, left; R, right; SNc, substantia nigra compacta; SN, substant [file 12974_2023_2709_MOESM1_ESM.pdf]

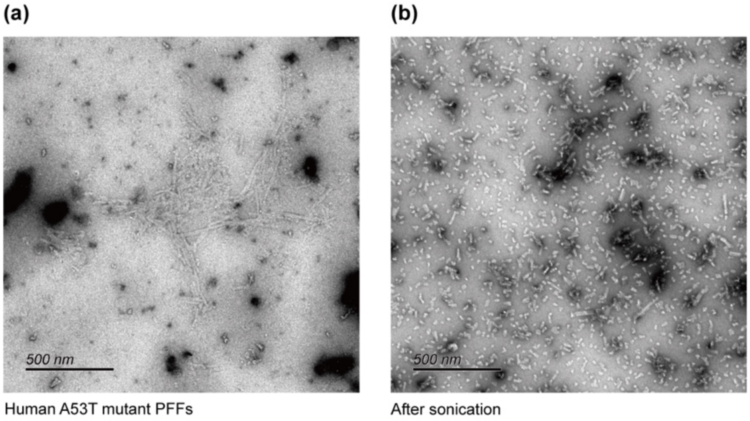

Supplementary Figure 1 Preparation of sonicated α-synuclein PFFs for stereotaxic injections. **Related to Fig. 1.**

(**a, b**) Representative TEM images of recombinant human A53T mutant α-synuclein PFFs before and after sonication. Scale bar: 500 nm.

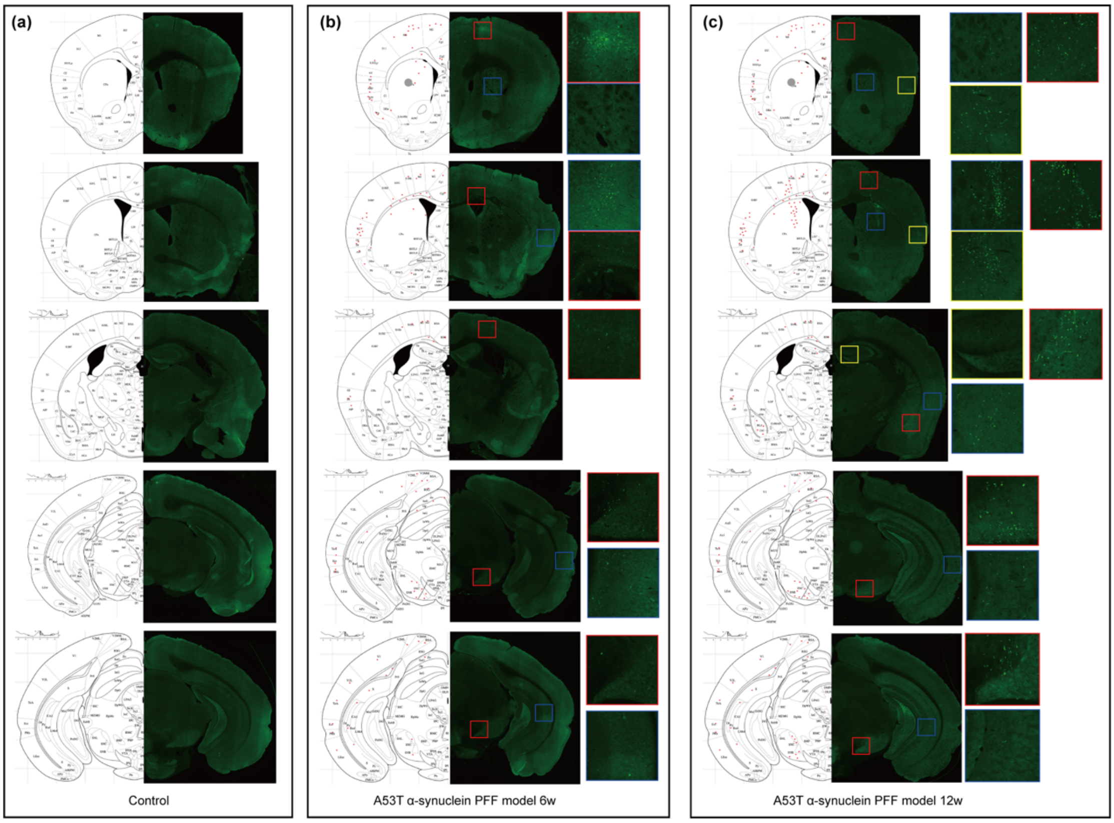

Supplementary Figure 2 Propagation of α-synuclein pathology in the mice brains as PD progresses. **Related to Fig. 1.**

(**a-c**) Representative immunofluorescence images of P-α-syn (green) in several brain slices of control

mice and PFFs-induced PD mice after six- and twelve- weeks treatment. The colored boxes in images are magnified on the right.

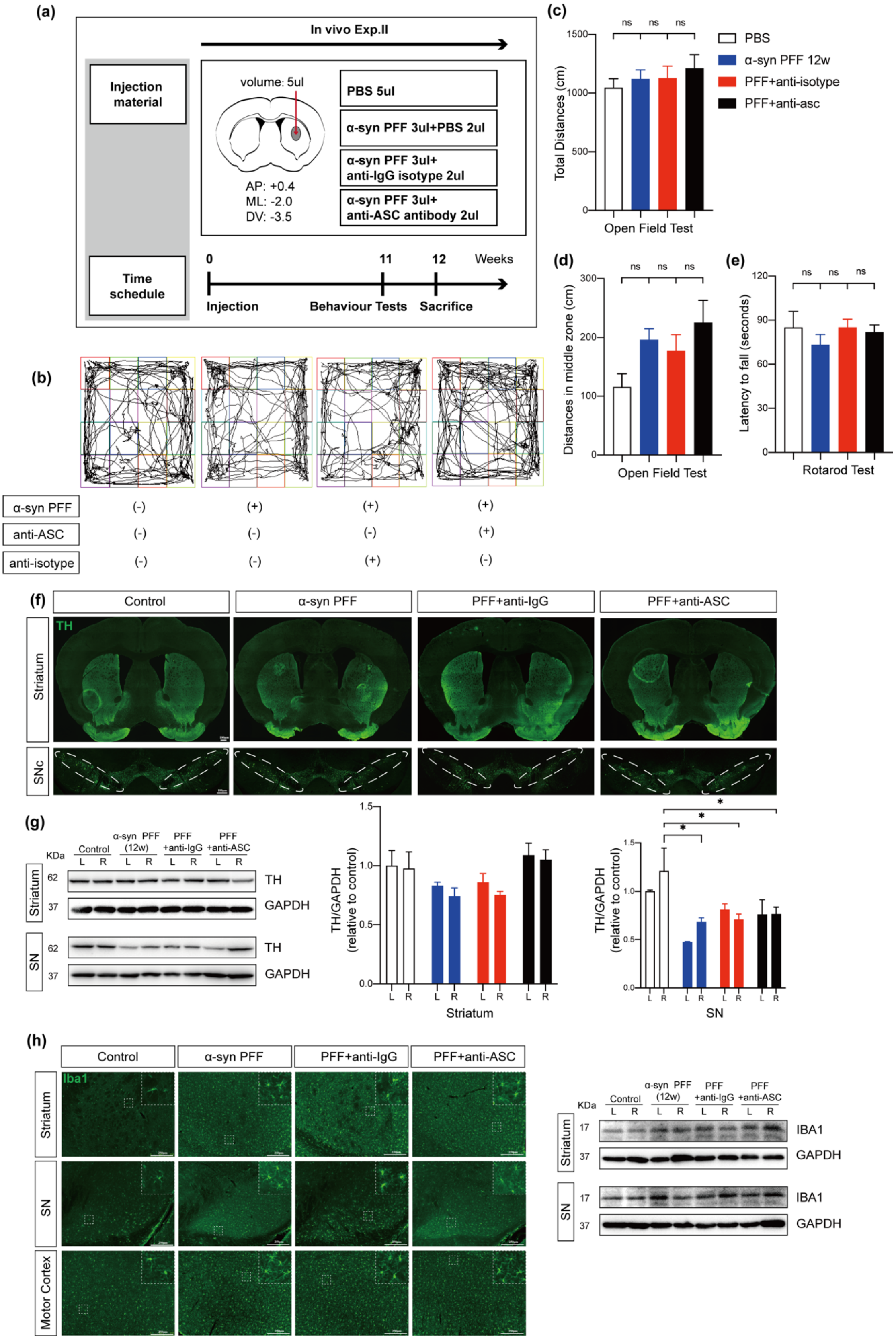

Supplementary Figure 3 No significant protective effects of anti-ASC antibody were found in PFFs-treated PD mice. **Related to Fig. 7.**

(**a**) Time schedules of the *in vivo* experiment in this study. The number of mice in each group: control group, n = 9; PFF group, n = 9; PFF+anti-IgG group, n = 12; PFF+anti-ASC group, n = 10. (**b**) Representative images of the tracks of indicated mice in open field test. (**c, d**) The total distance traveled and distance traveled in middle zone were recorded and analyzed for each mouse. (**e**) The latency to falling during the rotarod test was recorded and analyzed for each mouse. (**f**) Representative immunofluorescence staining of TH (green) neurons in the striatum and SNc of mice brains (n = 3-6). White dotted circles indicate the SNc regions. (**g**) IB analysis and quantification of TH in bilateral striatum and SN of indicated mice (n = 3). (**h**) Representative immunofluorescence staining of Iba1 (green) in the ipsilateral striatum, SN and motor cortex of mice brains (n = 3-6). White dotted squares show the morphology of microglia after magnification. (**i**) IB analysis of Iba1 in bilateral striatum and SN of indicated mice (n = 3). Values are presented as mean ± SEM and are analyzed by one-way or two-way ANOVA followed by Tukey's post hoc test for multiple comparisons. Levels of significance are: $*p < 0.05$; ns, no significant. Scale bars are as indicated. L, left; R, right; SNc, substantia nigra compacta; SN, substantia nigra.

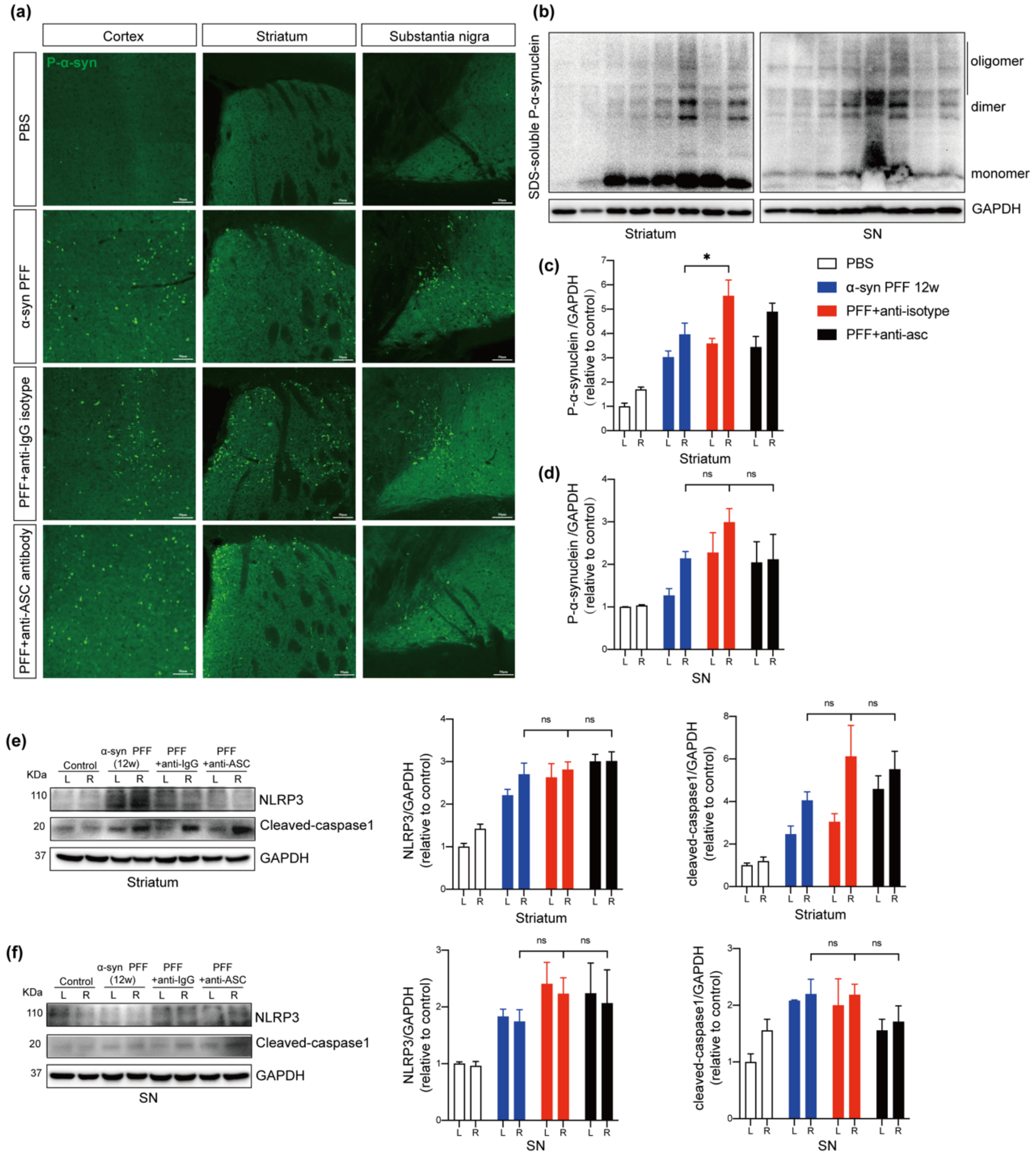

Supplementary Figure 4 No differences in α-synuclein pathology and NLRP3 inflammasome activation were observed in brain tissues of anti-ASC antibody treated or anti-isotype treated PD mice. **Related to Fig. 7.**

(**a**) Representative immunofluorescence staining of p-α-syn (green) in ipsilateral striatum, SN and cortex of indicated mice (n = 3-6). (**b-d**) IB analysis and quantification of SDS-soluble p-α-syn in bilateral striatum and SN tissues (n = 3). (**e, f**) IB analysis and quantification of NLRP3 inflammasome signals in bilateral striatum and SN of indicated mice (n = 3). Values are presented as mean ± SEM and are analyzed by two-way ANOVA followed by Tukey's post hoc test for multiple comparisons. Levels of significance are indicated as follows: *$p < 0.05$; ns, no significant. Scale bar: 50μm. L, left; R, right; SN, substantia nigra.
